# Reexamining Novel Risk Factors for Community-Acquired *Clostridioides difficile*: A Multimodal Approach

**DOI:** 10.1093/ofid/ofag598

**Published:** 2026-09-21

**Authors:** Nicholas A Turner, Angela Harris, Benjamin J Clark, Bobby G Warren, Aaron Barrett, Judith Kays, Deverick J Anderson

**Affiliations:** Division of Infectious Diseases, Duke University Medical Center, Durham, North Carolina, USA; Duke Center for Antimicrobial Stewardship and Infection Prevention, Duke University, Durham, North Carolina, USA; Department of Civil, Construction, and Environmental Engineering, NC State University, Raleigh, North Carolina, USA; Department of Civil, Construction, and Environmental Engineering, NC State University, Raleigh, North Carolina, USA; Duke Center for Antimicrobial Stewardship and Infection Prevention, Duke University, Durham, North Carolina, USA; Duke Center for Antimicrobial Stewardship and Infection Prevention, Duke University, Durham, North Carolina, USA; Department of Civil, Construction, and Environmental Engineering, NC State University, Raleigh, North Carolina, USA; Division of Infectious Diseases, Duke University Medical Center, Durham, North Carolina, USA; Duke Center for Antimicrobial Stewardship and Infection Prevention, Duke University, Durham, North Carolina, USA

**Keywords:** *C difficile*, community-acquired, livestock, surface water, wastewater

## Abstract

**Background:**

Geospatial surveys have identified livestock operations as potential sources for community-acquired *Clostridioides difficile* infection. Confirmatory studies integrating individual-level risk factors, geospatial data, and environmental surveys are limited.

**Methods:**

We conducted 2 parallel studies: (1) a case-control assessment of *C difficile* risk associated with livestock and (2) a microbiologic survey of surface waters and wastewater influent for *C difficile*. For aim 1, adult residents of North Carolina tested for *C difficile* through the Duke University Health System from March 2020 to December 2025 were included. Tests sent after day 3 of hospitalization (presumed hospital-onset) were excluded. Multivariable logistic regression with generalized estimating equations was used to assess *C difficile* risk associated with livestock operations while adjusting for age, comorbidities, antibiotic receipt, and hospital contact. For aim 2, samples collected from 3 surface water and 2 municipal wastewater sites in North Carolina from April 2022 to October 2023 were tested for *C difficile* gene targets by quantitative polymerase chain reaction.

**Results:**

The case-control cohort included 26 195 tests from 21 045 unique subjects, 3835 (14.6%) of which were positive for *C difficile* by nucleic acid amplification testing. Residence in the same watershed as commercial swine operations was associated with *C difficile* risk (adjusted odds ratio, 1.11 [95% confidence interval, 1.00–1.23]; *P* = .04). While *C difficile* gene targets were ubiquitous in municipal wastewater, they were not detected in 3 regional surface waters.

**Conclusions:**

Swine operations were associated with *C difficile* risk even after adjustment for individual-level risk factors. *Clostridioides difficile* detection in water varies by source.

While rates of hospital-onset *Clostridioides difficile* infection have modestly declined since 2011, rates of community-acquired *C difficile* remain largely unchanged [1]. As a result, community-acquired *C difficile* now comprises the majority of total cases presenting to care in some parts of the United States [2]. Prevention efforts are hindered by a limited understanding of the sources contributing to *C difficile* transmission in the community.

Emerging evidence suggests that animal and environmental sources may serve as reservoirs for *C difficile* acquisition in the community. Microbiologic surveys have confirmed high rates of environmental contamination at farms, abattoirs, in soil, and within a range of water sources [3–8]. Prior geospatial epidemiologic surveys—including one in North Carolina—have also identified proximity to livestock operations as a potential risk factor for community-acquired *C difficile* infection in humans [9]. There is even emerging molecular epidemiologic evidence confirming transmission of identical *C difficile* strain types between swine and humans [10]. Exact routes of transmission remain poorly understood, with hypotheses ranging from direct transmission between animals and agricultural workers, to contamination of food products, and even to dissemination via surface waters or air [11].

To confirm and refine prior geospatial associations between livestock operations and community-acquired *C difficile*, we undertook a multimodal study approach. North Carolina provides a unique opportunity for assessing animal and environmental risk factors as livestock operations are discretely distributed with cattle in the west, limited poultry operations in the east, and swine (the predominant livestock overall) distributed most densely in the southeastern portion of the state. First, we conducted a case-control study incorporating both livestock exposures and individual-level risk factors that might have served as key unaddressed confounders in prior research. Second, we conducted a microbiologic survey of surface waters to assess for environmental contamination with *C difficile*.

## MATERIALS AND METHODS

### Aim 1: Case-Control Study

#### Clinical Data Collection

For aim 1, we constructed a cohort of adult residents of North Carolina undergoing *C difficile* testing within the Duke University Health System, including 3 hospitals and their affiliated outpatient clinics, between 15 March 2020 and 31 December 2025. Study design was reviewed by the Duke University Health System's Institutional Review Board and determined to be exempt with waiver of consent for data collection.

To assess community acquisition of *C difficile*, cases were defined by a positive nucleic acid amplification test (NAAT). While our institution uses 2-step testing clinically, we opted to use a positive NAAT test as acquisition of *C difficile* was the outcome of interest (inclusive of both colonization and disease). Controls were defined by a negative NAAT result. Any tests collected from inpatients after hospital day 3 or following transfer from another healthcare facility were excluded as being presumably hospital or healthcare facility acquired (consistent with Centers for Disease Control and Prevention epidemiologic definitions) [12]. Age at time of testing, comorbidities present prior to testing, antibiotic receipt within 30 days prior to testing, and inpatient healthcare encounters within 90 days prior to testing were electronically queried across our entire health system.

The unit of analysis was by unique *C difficile* test result. As age, comorbidities, antibiotic receipt, home address, and healthcare contact were all treated as time-varying variables, >1 test could be included for individuals who underwent repeat testing so long as the tests were separated in time by at least 2 weeks and 1 or more individual-level variable changed between tests (eg, updated address, new comorbidity, new antibiotic receipt).

#### Geospatial Data Collection

Coordinates for each subject's confirmed address at time of testing were used to assign geospatial risk factors. Key geospatial risk factors of interest included the presence of commercial livestock operations within the same watershed as a patient's residence. Prior research has correlated the presence of livestock operations with microbial contamination of surrounding watersheds [13]. The location of commercial livestock operations and associated lagoons were obtained from the North Carolina Department of Environmental Quality [14]. Presence of a livestock operation within watersheds was determined by cross-referencing permit locations, patient address coordinates, and the United States Geological Survey watershed maps (accessed via the streamstats package in R and spatially merged using the sf package) [15, 16]. Population density was obtained from the 2021 American Community Survey, accessed via the tigris package in R, and calculated at the census tract level [17].

#### Statistical Modeling

To account for the fact that some patients could contribute >1 test result to the cohort, we used a generalized estimating equation approach clustered by patient identifier. Generalized estimating equations offer more robust assessment of confidence intervals (CIs) when there is violation of independence between tests [18]. Logistic regression models were then constructed using both individual-level (age, comorbidities, high-risk antibiotic receipt, inpatient encounter within the preceding 90 days) and geospatial (presence of a commercial animal operation within watershed) risk factors. High-risk antibiotics were defined as fluoroquinolones, third- or fourth-generation cephalosporins, clindamycin, and carbapenems. Potential for confounding was addressed by including consistently identified risk factors for *C difficile* in all multivariable analyses, including age, high-risk antibiotic receipt, hospital contact, chronic kidney disease, and cancer [19–21]. All numerical variables were assessed for potential violations of linearity assumption by testing against restricted cubic splines. Variables with evidence of nonlinearity were transformed as appropriate. All multivariable models were assessed for potential collinearity by examining variance inflation factors; none met criteria for exclusion. All models were constructed using geepack in R (version 4.1.2, https://cran.r.project.org) [22]. A *P* value of .05 was used to assess significance. As all other analyses were either descriptive or exploratory, no other *P* values are reported.

As model interpretation hinges heavily on modeling assumptions, we conducted multiple sensitivity analyses to assure model stability across various de-duplication strategies, handling of individual-level risk factors, and consideration of additional geospatial risk factors.

### Aim 2: Microbiologic Survey

#### Wastewater Filtration and DNA Extraction

In the accompanying microbiologic survey, sewage influent samples were collected 2 municipal wastewater treatment plants between April 2022 and October 2023 (Supplementary Figure 1). Wastewater samples were collected in 250-mL sterile polypropylene containers and stored on ice or at 4°C prior to processing. Wastewater samples had 40 mL membrane-filtered through 0.45-µm HA filters (Millipore, Burlington, MA, USA) after being spiked with MgCl_2_ to a final concentration of 25 mM and bovine rota-coronavirus vaccine used as an internal amplification control (modified-live virus) (Zoetis, Parsippany, NJ, USA). Filters were spiked with 100 000 copies of VetMax Xeno Internal Positive DNA Control (Applied Biosystems, Foster City, CA, USA) to assess extraction efficiency, and nucleic acids were extracted from the filters using a modified version of the Qiagen RNeasy Powerfecal Pro Kit (SI) to co-extract DNA and RNA. Nucleic acids were quantified with NanoDrop.

#### Surface Water Filtration and DNA Extraction

Surface water samples were collected from 3 flowing streams in eastern North Carolina. The surface water samples were collected in 1-L sterile polypropylene containers and stored on ice prior to processing. Surface waters had 80 mL of sample vacuum-filtered through 0.4-µm Isopore filters (Millipore). Nucleic acids were extracted using Qiagen's DNeasy Powersoil Pro DNA extraction kit with the Qiacube HT and quantified with NanoDrop.

#### Quantification of *C difficile* Gene Targets in Wastewater

*Clostridioides difficile* was assessed by quantitative polymerase chain reaction (qPCR) for 2 gene targets: glutamate dehydrogenase (*gluD*, which is highly conserved across *C difficile* strains) and toxin B (*tcdB*, which is specific to toxigenic *C difficile* strains) [23–25]. qPCR for 99 wastewater samples was conducted using a 30-µL reaction mixture that contained 15 µL TaqMan Environmental Master Mix 2.0, 2 µL template, forward and reverse primers to a final concentration of 900 nM, probe to a final concentration of 250 nM, bovine serum albumin to a final concentration of 0.2 mg/mL, and molecular-grade water to achieve a final reaction volume of 30 µL. Thermocycling included enzyme activation at 95°C for 10 minutes followed by 40 cycles of denaturation at 95°C for 15 seconds and annealing and extension at 60°C for 1 minute. A standard curve was constructed with serial dilutions from 200 000 copies per reaction to 20 copies per reaction. The standard curve was constructed with linear regression using R. Samples were run in triplicate where a positive sample would be determined if 2 of 3 replicates amplified. There were 4 no-template controls that returned negative results. All samples had the concentration of Xeno internal positive DNA control assessed with a custom-made TaqMan Array card and compared to Xeno concentrations in extraction blanks, yielding no evidence of inhibition from the wastewater matrix. There were 5 extraction blanks and 8 filtration blanks that were tested and were free from contamination.

#### Quantification of *C difficile* Gene Targets in Surface Water

*Clostridioides difficile* was assessed by qPCR for 1 gene target: glutamate dehydrogenase (*gluD*). qPCR for 149 surface water samples was conducted using 10 µL of TaqMan Fast Advanced Master Mix for qPCR, 2 µL of template, forward and reverse primers to a final concentration of 900 nM, probe to a final concentration of 250 nM, and molecular-grade water to achieve a final reaction volume of 20 µL. Thermocycling included enzyme activation at 95°C for 20 seconds followed by 40 cycles of denaturation at 95°C for 1 second and annealing and extension at 60°C for 20 seconds. A standard curve was constructed with serial dilutions from 2 000 000 copies per reaction to 20 copies per reaction. The standard curve was constructed with linear regression using R. Samples were run in triplicate where a positive sample would be determined if 2 of 3 replicates amplified. There were 13 no-template controls that were free from contamination. Twenty percent (30/149) of samples underwent inhibition testing by spiking 2000 gene copies into a single comparison reaction and were found to not be inhibited. There were 3 extraction blanks and 27 field blanks that were tested and were free from contamination.

## RESULTS

### Aim 1: Case-Control Study

The case-control cohort included 26 195 tests collected from 21 045 unique subjects from March 2020 through December 2025 (Figure 1). In the overall cohort, 3835 (14.6%) tests were NAAT positive (cases) and 22 360 (85.4%) tests were NAAT negative (controls). Cohort demographics, comorbidities, antibiotic receipt, healthcare facility contact, and geospatial variables are summarized in Table 1. Median age was numerically higher among cases relative to controls. Several key comorbidities were also relatively more frequent among cases than controls, reflected in the slightly higher median Charlson Comorbidity Index score among cases. High-risk antibiotic receipt and admission in the preceding 90 days were also numerically more prevalent among cases than controls.

**Figure1.**
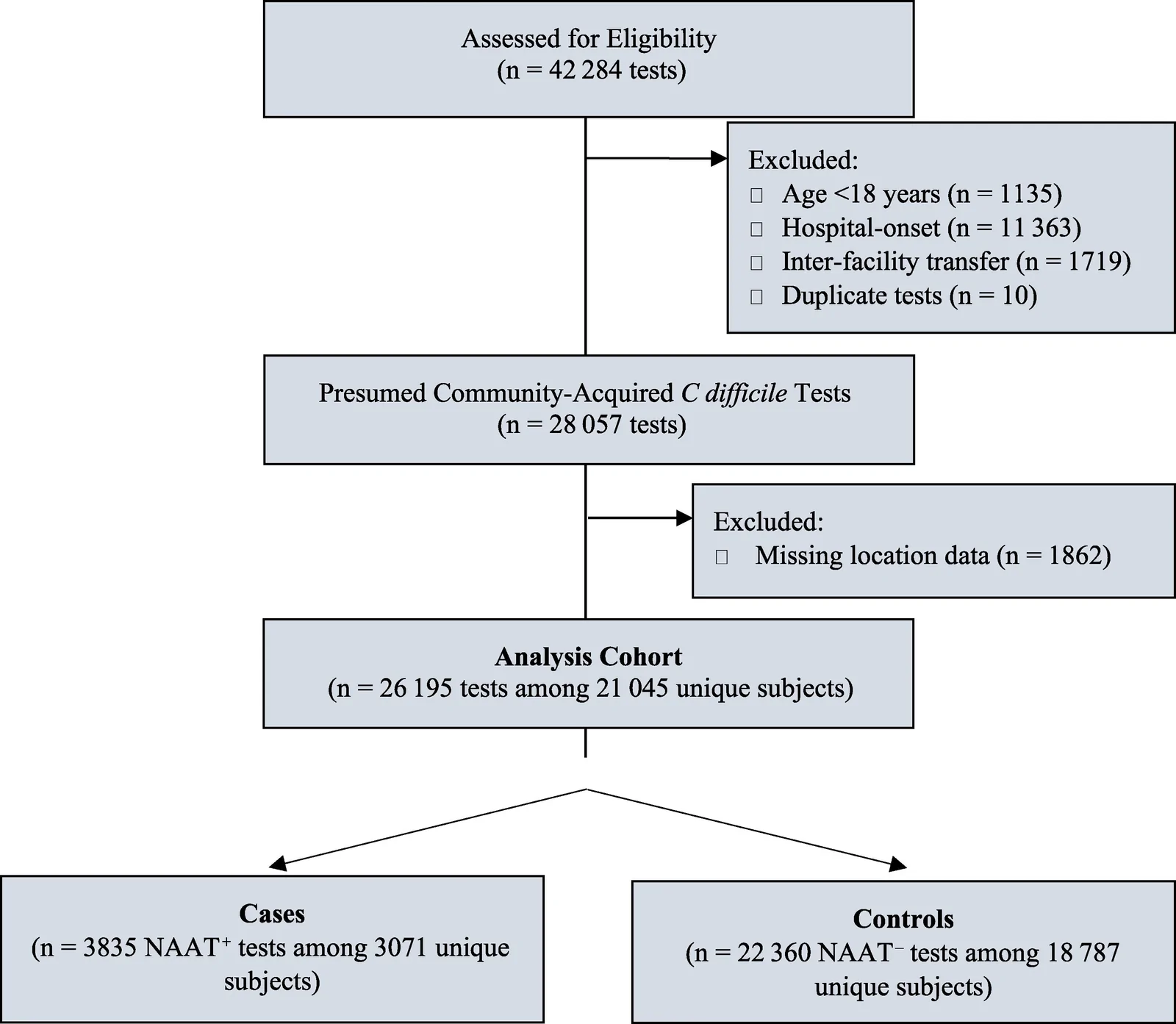
Consolidated Standards of Reporting Trials (CONSORT)–style diagram. Since subjects could have had more than one test, unique subjects could appear in both the cases and controls. Unit of analysis was by test. Abbreviation: NAAT, nucleic acid amplification test.

**Table 1.** Cohort Characteristics

Characteristic	Full Cohort (N = 26 195 Tests)	Cases (NAAT Positive) (n = 3835 Tests)	Controls (NAAT Negative) (n = 22 360 Tests)
Age, y, median (IQR)	61.2 (44.3–72.6)	63.7 (47.8–74.4)	60.6 (43.8–72.3)
Sex, female	16 209 (61.9)	2464 (64.3)	13 745 (61.5)
Race			
Black or African American	6104 (23.3)	994 (25.9)	5110 (22.9)
White	18 082 (69.0)	2621 (68.3)	15 461 (69.1)
Asian	654 (2.5)	75 (2.0)	579 (2.6)
Other	787 (3.0)	79 (2.1)	708 (3.2)
Not reported	568 (2.2)	66 (1.7)	502 (2.2)
Ethnicity			
Hispanic/Latino	1314 (5.0)	129 (3.4)	1185 (5.3)
Non-Hispanic/Latino	24 017 (91.7)	3585 (93.5)	20 432 (91.4)
Not reported	864 (3.3)	121 (3.2)	743 (3.3)
Comorbidities			
Complicated diabetes	597 (2.3)	126 (3.3)	471 (2.1)
Chronic kidney disease	7763 (29.6)	1499 (39.1)	6264 (28.0)
Cancer	7089 (27.1)	1242 (32.4)	5847 (26.1)
Liver disease	4800 (18.3)	881 (23.0)	3919 (17.5)
Solid organ transplant	1620 (6.2)	312 (8.1)	1308 (5.8)
CCI score, median (IQR)	2.0 (0.0–5.0)	3.0 (1.0–6.0)	2.0 (0.0–4.0)
High-risk antibiotics	5179 (21.7)	1139 (29.7)	4886 (21.9)
Positive *C difficile* test in preceding 90 d	681 (2.6)	450 (11.7)	231 (1.0)
Admission in preceding 90 d	16 349 (62.4)	2786 (72.6)	13 563 (60.7)
Population density, median (IQR)	1376 (378–2776)	1155 (309–2497)	1383 (382–2789)
Presence of commercial swine operation in same watershed	6758 (25.8)	1048 (27.3)	5710 (25.5)
Presence of commercial cattle operation in same watershed	2188 (8.4)	336 (8.8)	1852 (8.3)
Presence of commercial poultry operation in same watershed	332 (1.3)	54 (1.4)	278 (1.2)

Abbreviations: CCI, Charlson Comorbidity Index; IQR, interquartile range; NAAT, nucleic acid amplification test.

Test distribution relative to livestock exposure is mapped in Figure 2. Sampling was heterogenous across the state, with the highest concentration of tests in central North Carolina where Duke Health's main campuses are located (Figure 2*A*). The number of commercial swine operations per watershed was highest in the southeastern portion of the state where sampling was slightly less robust—though several key counties contributed >50 tests each and just over 25% of the total cohort resided within the same watershed as a commercial swine operation (Figure 2*B*). Other livestock operations were more limited in distribution, and patient sampling near either cattle or poultry operations was sparser as a result (Figure 2*C* and 2*D*).

**Figure 2.**
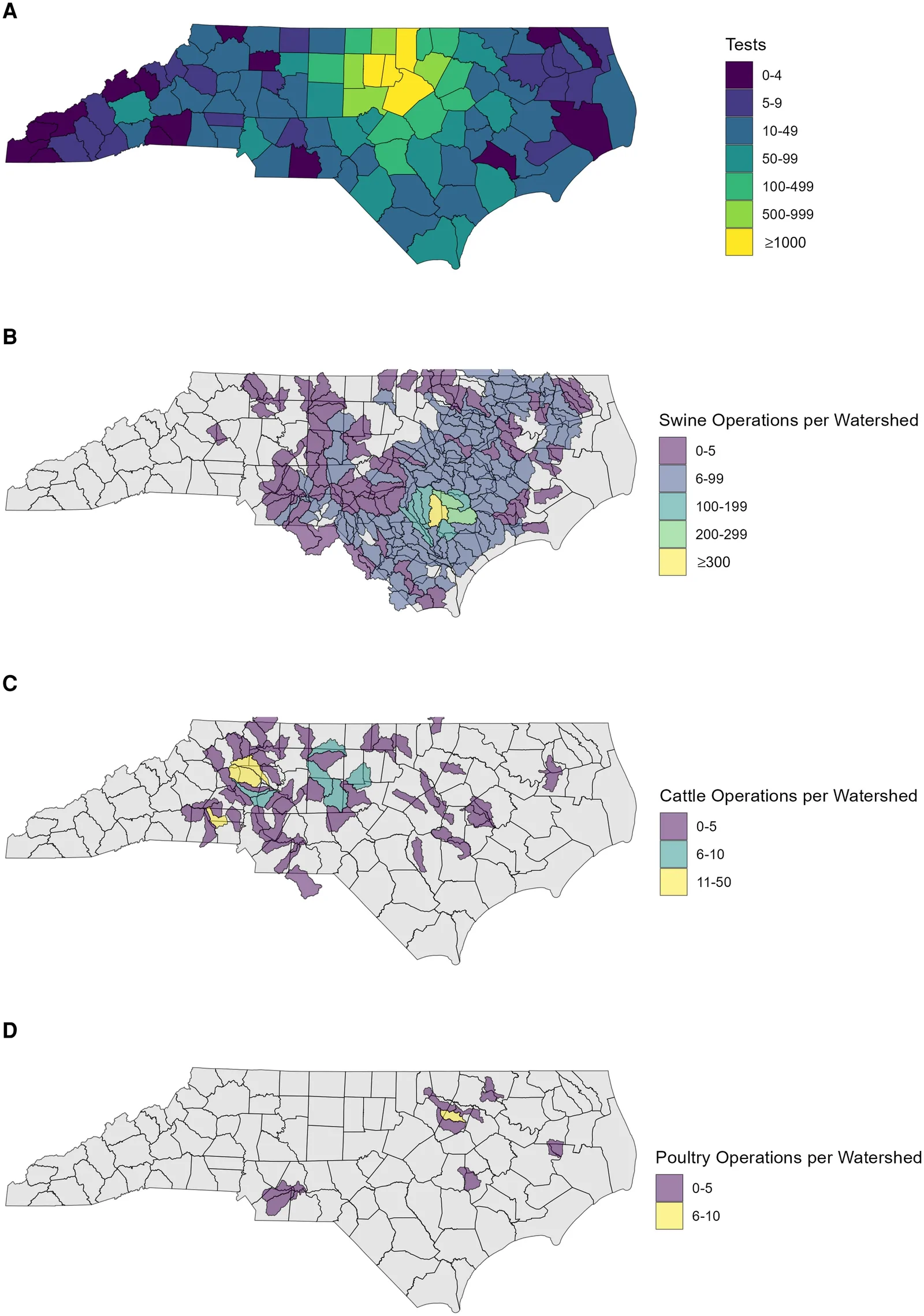
Maps of test distribution by county and livestock operations by watershed. The base map in each case is North Carolina with counties delineated. *A*, Distribution of *Clostridioides difficile* tests included in the study cohort as color coding by county. *B*, Overlay of watersheds color coded by number of swine operations contained within. *C*, Overlay of watersheds color coded by cattle operations contained within. *D*, Overlay of watersheds color coded by poultry operations contained within.

In the primary analysis, swine operations were positively associated with *C difficile* risk even after adjustment for individual-level risk factors (adjusted odds ratio [aOR], 1.11 [95% CI, 1.00–1.23]; *P* = .04). Neither cattle (aOR, 0.98 [95% CI, .84–1.14]; *P* = .79) nor poultry (aOR, 1.05 [95% CI, .76–1.45]; *P* = .76) exhibited any association with *C difficile* risk; however, CIs were wide due to sparse sampling (Table 2). The previously described individual-level risk factors for *C difficile*—which were included in the model as potential confounders—all exhibited the expected positive risk associations consistent with prior literature [19]. Adjustment for individual-level confounders modestly mitigated the risk associated with swine operations: The odds ratio (OR) decreased only slightly from 1.16 (95% CI, 1.07–1.27) on univariate analysis to 1.11 (95% CI, 1.00–1.23) on adjusted analysis.

**Table 2.** Risk Modeling for Community-Acquired *Clostridioides difficile* Infection

Covariate	Univariate (Unadjusted) OR (95% CI)	Multivariate (Adjusted) OR (95% CI)
Age, per 10 y	1.09 (1.07–1.12)	1.04 (1.01–1.06)^a^
High-risk antibiotic receipt	1.36 (1.23–1.50)	1.25 (1.14–1.37)^a^
Prior *C difficile* within 90 d	1.35 (1.00–1.82)	1.43 (1.10–1.87)^a^
Cancer	1.36 (1.25–1.49)	1.11 (1.01–1.22)^a^
Chronic kidney disease	1.59 (1.45–1.72)	1.36 (1.25–1.49)^a^
Admission in preceding 90 d	1.55 (1.42–1.69)	1.33 (1.22–1.47)^a^
Swine operation in watershed	1.16 (1.07–1.27)	1.11 (1.00–1.23)^a^
Cattle operation in watershed	1.10 (0.96–1.26)	0.98 (.84–1.14)
Poultry operation in watershed	1.25 (.92–1.71)	1.05 (.76–1.45)

Abbreviations: CI, confidence interval; OR, odds ratio.

^a^Denotes associations reaching statistical significance (*P* < .05).

While the primary analysis used a generalized estimating equations approach to account for violation of independence (since subjects might have been tested more than once), we also conducted a series of sensitivity analyses to assess whether differing de-duplication rules might impact effect estimates. Across multiple different models using 3 different de-duplication rules, presence of a commercial swine operation within a watershed remained associated with *C difficile* risk: ORs ranged from 1.06 to 1.11, though in many cases the smaller numbers from more strictly de-duplicated cohorts resulted in the 95% CI crossing 1 (Supplementary Tables 1–3).

While particular livestock operations were the primary exposure of interest, it remains possible that other exposures common to rural or agricultural settings might still play a role in *C difficile* transmission. To assess this, we explored other potential markers of rurality in association with *C difficile* risk. While sample size severely constrained additional analyses, we did observe an inverse association between *C difficile* risk and population density (OR, 0.97 [95% CI, .95–.98] per decile; Supplementary Table 4). Inclusion of population density as an additional covariate did mitigate the association with swine operations such that an OR of 1 was no longer excluded from the 95% CI (OR, 1.07 [95% CI, .98–1.17]), though, of note, regions with lower population density tended to correlate with higher density of agricultural operations.

### Aim 2: Microbiologic Survey

We did not detect *C difficile* glutamate dehydrogenase (*gluD*) in any of the 149 surface water samples collected across 3 sites in southeastern North Carolina (Table 3). In contrast to surface waters, we were able to detect *gluD* and *tcdB* targets consistently from municipal wastewater (99 of 99 samples).

**Table 3.** Microbiologic Survey of Water Samples

Water Sampling Site	*gluD* Detections, no./No. (%)	*gluD* Copies/µL, Median (IQR)	*tcdB* Detections, no./No. (%)	*tcdB* Copies/µL, Median (IQR)
Surface water site A	0/50 (0)	0 (0–0)	0/50 (0)	0 (0–0)
Surface water site B	0/49 (0)	0 (0–0)	0/49 (0)	0 (0–0)
Surface water site C	0/50 (0)	0 (0–0)	0/50 (0)	0 (0–0)
Wastewater treatment plant A	47/47 (100)	68 (40–119)	47/47 (100)	42 (30–62)
Wastewater treatment plant B	52/52 (100)	33 (25–59)	52/52 (100)	22 (15–30)

Abbreviations: IQR, interquartile range; *gluD*, *Clostridioides difficile* glutamate dehydrogenase gene; *tcdB*, *Clostridioides difficile* toxin B gene.

## DISCUSSION

In this multimodal investigation of risk factors for community-acquired *C difficile* within North Carolina, residing in the same watershed as a commercial swine operation was associated with *C difficile* risk even after adjustment for individual-level confounders. Sparser sampling limited assessment of other livestock types. Despite the apparent association between swine operations and *C difficile* risk, we were unable to detect *C difficile* gene targets from any of the 3 surface water sites sampled from a region of the state where swine operations are common.

Overall, our results corroborate prior geospatial studies suggesting an association between livestock operations and *C difficile* risk—though our study aimed to better delineate risk by specific livestock type. While Anderson et al previously found proximity to livestock operations overall to be associated with *C difficile* risk [9], that study was also conducted in North Carolina where swine represent the majority of livestock operations. In the present study, we used an updated cohort spanning a more recent time interval and larger area of the state to better capture variation in specific livestock exposures as these tend to cluster in different regions of our state (Figure 2). The particular association that we observed with swine operations has also been observed previously—including molecular epidemiologic studies confirming the same *C difficile* strains in swine as in human subjects [10]. The current study cannot be considered confirmatory, however. While the observed association with *C difficile* seemed specific to swine, sparse sampling limits the confidence of our risk estimates for cattle or poultry operations. It also remains possible that an entirely different exposure route might be common to rural areas besides livestock operations. In an exploratory sensitivity analysis, for example, population density was both inversely associated with *C difficile* risk and mitigated the association with swine operations. Because swine operations also tend to be located in less populated regions of the state, parsing out specific risk factors that may be common to rural areas is not possible with the data available in this study. Ideally, more definitive studies would include prospective sampling at a larger scale and with better sampling across geographic variables of interest.

If the observation that swine operations contribute to *C difficile* transmission in the community is true, the exact routes of transmission remain unresolved. Several genomic epidemiology surveys have confirmed circulation of identical or nearly identical *C difficile* isolates between swine and humans [26, 27]. Swine have been of particular interest as a potential source, given high rates of colonization particularly among younger piglets [28–34]. While some studies have observed high rates of resistance to commonly used agricultural antibiotics among *C difficile* isolates from pigs, colonization also seems to occur frequently even in the absence of known antibiotic exposure [35, 36]. Other studies have found variants in trehalose metabolism—a feed supplement—to be associated with increased *C difficile* virulence, suggesting that food additives may also play a role in agriculture-associated *C difficile*. An array of hypotheses have been proposed for transmission from swine to humans, including contamination of meat products, transmission to agricultural workers, and even transmission via soil, water, or air, but none have been definitively proven [11, 28, 29]. While our environmental sampling was limited to just 2 municipal wastewater treatment plants and 3 surface waters, the 3 surface waters sampled were located in a portion of the state where swine operations are prevalent. The lack of *C difficile* detection in surface waters does not support waterborne transmission in the rural setting, but it also cannot rule out this possibility. It is possible, for example, that spores may sediment to the bottom of stiller water sources. Similarly, as *C difficile* is anaerobic, vegetative forms may proliferate better in the relatively anoxic sediments. Additionally, optimal sampling may differ for moving versus stagnant water. In this case, sampling either soil or sediment rather than the water column itself might improve detection. Further, *C difficile* may be present in the surface waters at concentrations below our assay limit of detection. Techniques to increase assay sensitivity and lower the limit of detection could be employed, such as increasing the sample volume processed (eg, dead-end ultrafiltration) and the use of passive samplers [37, 38]. Future microbiologic surveys could assess diverse sampling strategies to better inform optimal methods.

Overall, our study carries several strengths—most notably the reasonably large sample size spanning a geographic area with widely varying exposure to swine operations. In contrast to prior geospatial studies, we were also able to use electronically queried medical records to better adjust for individual-level confounders such as antibiotic receipt, comorbidities, and prior hospital admissions.

Out study still carries several key limitations, most critically that sampling remained heterogenous and was specifically less robust in more rural areas with highest livestock concentration. Given the heterogeneity in sampling, we could only incorporate geospatial risk factors as a modeled exposure—and could not adequately assess for any apparent case-clustering in regions of interest or assess other potential risk factors common to rural areas. The microbiologic survey carries similar limitations: While water sampling was undertaken in a region with a relatively high concentration of livestock operations, it was performed at just 3 sites and did not include any actual swine lagoons or agricultural wastewater directly. While we were able to detect *C difficile* from municipal wastewater, methods for testing *C difficile* in surface water are less well-validated. Finally, all of the reported work carries the potential for residual confounding inherent to any retrospectively conducted observational study.

In conclusion, we observed a similar risk association between livestock operations—specifically swine—and community-acquired *C difficile* risk to prior studies despite an inability to detect *C difficile* gene targets from regional surface waters. Confirmation of a link between livestock operations and *C difficile* risk will still require well-conducted, ideally prospectively enrolled, studies to overcome issues with small sample sizes and heterogenous sampling inherent to existing observational datasets.

## Supplementary Material

ofag598_Supplementary_Data

## Supplementary Data

Supplementary materials are available at *Open Forum Infectious Diseases* online. Consisting of data provided by the authors to benefit the reader, the posted materials are not copyedited and are the sole responsibility of the authors, so questions or comments should be addressed to the corresponding author.

## Notes

***Author contributions.*** N. A. T. was responsible for conceptualization, data curation, formal analysis, and writing—original draft. A. H. was responsible for conceptualization, data curation, formal analysis, and writing—review and editing. B. J. C. was responsible for formal analysis, investigation, and writing—review and editing. B. G. W. and A. B. were responsible for investigation, data curation, and writing—review and editing. J. K. was responsible for methodology. D. J. A. was responsible for conceptualization, supervision, funding acquisition, and writing—review and editing. All authors had full access to all the data in the study and take responsibility for the integrity of the data and the accuracy of the data analysis. The content of this manuscript is original, and has not been previously published.

***Patient consent.*** Study design was reviewed by Duke University Health System's Institutional Review Board and determined to be exempt with waiver of consent for data collection.

***Data availability.*** Data are not publicly available as they include geospatial identifiers. Select de-identified data can be made available upon request to corresponding author.

***Financial support.*** This work was supported by the Prevention Epicenters Program of the Centers for Disease Control and Prevention (U54CK000616, Principal Investigator D.J.A.) and an additional pilot grant from the Duke Clinical and Translational Science Institute. Surface water samples were collected as part of a project funded by the North Carolina Water Resources Research Institute.
