# Supplementary material for: Reexamining Novel Risk Factors for Community-Acquired Clostridioides difficile: A Multimodal Approach
Source: Open Forum Infect Dis. 2026 Sep 21;13(10):ofag598. doi: 10.1093/ofid/ofag598 (PMC13625192; doi:10.1093/ofid/ofag598)
Supplement: Supplementary file 1 [file ofag598_supplementary_data.docx]

**SUPPLEMENT**

**Figure S1. Maps of Water Sampling Sites.**

The base map in each case is a map of North Carolina with counties delineated. **Panel A** displays the three surface water sampling sites (represented by dark blue circles) with an overlay of relevant watersheds color-coded by number of swine operations contained within. **Panel B** displays the two municipal wastewater sampling sites (represented by light blue circles) with an overlay of watersheds color coded for the presence of a municipal wastewater plant contained within.

**Table S1. Risk Modeling for Community-Onset *C. difficile* Infection – Sensitivity Analysis 1: Varying Adjustments for Comorbidities.**

| Covariate | Univariate OR (95% CI) | Maximally Adjusted Multivariate OR (95% CI) | CCMI^a^ Adjusted Multivariate OR (95% CI) | Literature Informed Multivariate OR (95% CI) | Parsimonious^b^ Multivariate OR (95% CI) |
| --- | --- | --- | --- | --- | --- |
| Age, per 10 years | 1.09 (1.07-1.12) | 1.02 (1.00-1.05) | 1.05 (1.02-1.07) | 1.04 (1.01-1.06) | -- |
| Any high-risk antibiotic receipt | 1.36 (1.23-1.50) | 1.24 (1.14-1.36) | 1.24 (1.13-1.36) | 1.25 (1.15-1.37) | 1.24 (1.13-1.36) |
| Prior *C. difficile* within 90 days | 1.35 (1.00-1.82) | 1.44 (1.11-1.88) | 1.39 (1.06-1.83) | 1.43 (1.10-1.87) | 1.43 (1.09-1.86) |
| Cancer | 1.36 (1.25-1.49) | 1.08 (0.98-1.20) | -- | 1.11 (1.01-1.22) | 1.10 (1.00-1.22) |
| Chronic kidney disease | 1.58 (1.45-1.72) | 1.24 (1.12-1.36) | -- | 1.37 (1.25-1.49) | 1.26 (1.15-1.38) |
| Complicated diabetes | 1.61 (1.25-2.08) | 1.26 (0.99-1.61) | -- | -- | 1.24 (0.98-1.58) |
| Congestive heart failure | 1.58 (1.45-1.73) | 1.23 (1.11-1.35) | -- | -- | 1.24 (1.13-1.37) |
| Connective tissue disease | 1.10 (0.95-1.28) | 1.01 (0.87-1.17) | -- | -- | -- |
| Dementia | 1.62 (1.31-2.01) | 1.40 (1.14-1.71) | -- | -- | 1.44 (1.17-1.75) |
| HIV | 0.63 (0.42-0.93) | 0.57 (0.39-0.85) | -- | -- | 0.57 (0.38-0.84) |
| Liver | 1.35 (1.21-1.50) | 1.03 (0.92-1.16) | -- | -- | -- |
| Severe liver | 1.80 (1.53-2.13) | 1.41 (1.17-1.69) | -- | -- | 1.43 (1.21-1.68) |
| Pulmonary | 1.18 (1.08-1.28) | 1.04 (0.95-1.13) | -- | -- | 1.04 (0.96-1.13) |
| Paresis | 1.54 (1.24-1.92) | 1.23 (1.01-1.51) | -- | -- | 1.23 (1.01-1.51) |
| Metastatic | 1.42 (1.24-1.61) | 1.12 (0.98-1.29) | -- | -- | 1.13 (0.98-1.30) |
| Solid organ transplant | 1.44 (1.23-1.69) | 1.00 (0.84-1.19) | -- | -- | -- |
| Charlson co-morbidity index | 1.07 (1.06-1.08) | -- | 1.05 (1.03-1.06) | -- | -- |
| Admission in preceding 90 days | 1.55 (1.42-1.69) | 1.27 (1.15-1.40) | 1.30 (1.18-1.43) | 1.34 (1.22-1.47) | 1.27 (1.16-1.40) |
| Swine CAFO present | 1.16 (1.07-1.27) | 1.10 (1.01-1.20) | 1.10 (1.01-1.20) | 1.11 (1.02-1.21) | 1.09 (1.00-1.19) |
| ^a^Charlson comorbidity index.  ^b^Parsimonious model developed by stepwise selection using Akaike information criterion.[1] | | | | | |

**Table S2. Risk Modeling for Community-Onset *C. difficile* Infection – Sensitivity Analysis 2:**

**Exclusion of Any CDI in Preceding 90 days**

| Covariate | Maximally Adjusted Multivariate OR (95% CI) | CCMI^a^ Adjusted Multivariate OR (95% CI) | Literature Informed Multivariate OR (95% CI) | Parsimonious^b^  Multivariate OR (95% CI) |
| --- | --- | --- | --- | --- |
| Age, per 10 years | 1.01 (0.99-1.04) | 1.04 (1.02-1.07) | 1.03 (1.01-1.06) | -- |
| Any high-risk antibiotic receipt | 1.31 (1.20-1.43) | 1.31 (1.20-1.42) | 1.32 (1.21-1.44) | 1.31 (1.20-1.43) |
| Cancer | 1.07 (0.97-1.18) | -- | 1.10 (1.00-1.20) | 1.09 (0.99-1.20) |
| Chronic kidney disease | 1.23 (1.12-1.35) | -- | 1.37 (1.25-1.49) | 1.26 (1.15-1.38) |
| Complicated diabetes | 1.23 (0.97-1.56) | -- | -- | 1.21 (0.96-1.53) |
| Congestive heart failure | 1.22 (1.11-1.34) | -- | -- | 1.23 (1.12-1.36) |
| Connective tissue disease | 1.04 (0.90-1.20) | -- | -- | -- |
| Dementia | 1.46 (1.19-1.78) | -- | -- | 1.48 (1.22-1.80) |
| HIV | 0.58 (0.40-0.87) | -- | -- | 0.57 (0.39-0.84) |
| Liver | 1.05 (0.94-1.17) | -- | -- | -- |
| Severe liver | 1.30 (1.09-1.56) | -- | -- | 1.36 (1.16-1.59) |
| Pulmonary | 1.04 (0.96-1.13) | -- | -- | 1.05 (0.97-1.14) |
| Paresis | 1.22 (1.00-1.49) | -- | -- | 1.22 (1.00-1.48) |
| Metastatic | 1.13 (0.98-1.29) | -- | -- | 1.13 (1.00-1.30) |
| SOT | 1.06 (0.89-1.24) | -- | -- | -- |
| Charlson index | -- | 1.04 (1.03-1.06) | -- | -- |
| Admission in preceding 90 days | 1.27 (1.16-1.39) | 1.31 (1.20-1.44) | 1.35 (1.24-1.48) | 1.28 (1.17-1.40) |
| Swine CAFO present | 1.06 (0.96-1.16) | 1.09 (1.00-1.19 | 1.10 (1.01-1.20) | 1.09 (1.00-1.19) |
| ^a^Charlson comorbidity index.  ^b^Parsimonious model developed by stepwise selection using Akaike information criterion.[1] | | | | |

**Table S3. Risk Modeling for Community-Onset *C. difficile* Infection – Sensitivity Analysis 3:**

**Simple Deduplication (Including only the first test for each subject)**

| Covariate | Univariate OR (95% CI) | Individually Adjusted Multivariate OR (95% CI) | CCMI Adjusted Multivariate OR (95% CI) | Literate Driven Multivariate OR (95% CI) | Parsimonious Multivariate OR (95% CI) |
| --- | --- | --- | --- | --- | --- |
| Age, per 10 years | 1.09 (1.07-1.11) | 1.01 (0.98-1.03) | 1.03 (1.01-1.05) | 1.02 (1.00-1.05) | -- |
| Any high-risk antibiotic receipt | 1.79 (1.64-1.94) | 1.45 (1.32-1.58) | 1.45 (1.33-1.58) | 1.46 (1.33-1.59) | 1.45 (1.32-1.58) |
| Cancer | 1.43 (1.32-1.56) | 1.11 (1.00-1.23) | -- | 1.13 (1.03-1.23) | 1.12 (1.01-1.23) |
| Chronic kidney disease | 1.74 (1.60-1.88) | 1.28 (1.14-1.41) | -- | 1.45 (1.33-1.59) | 1.32 (1.20-1.44) |
| Complicated diabetes | 1.81 (1.42-2.30) | 1.30 (1.01-1.67) | -- | -- | 1.31 (1.02-1.68) |
| Congestive heart failure | 1.69 (1.55-1.84) | 1.23 (1.11-1.36) | -- | -- | 1.24 (1.12-1.37) |
| Connective tissue disease | 1.23 (1.06-1.42) | 1.09 (0.94-1.26) | -- | -- | -- |
| Dementia | 1.66 (1.36-2.02) | 1.39 (1.13-1.71) | -- | -- | 1.40 (1.14-1.70) |
| HIV | 0.66 (0.44-0.94) | 0.57 (0.38-0.83) | -- | -- | 0.57 (0.38-0.82) |
| Liver | 1.46 (1.33-1.61) | 1.06 (0.95-1.19) | -- | -- | -- |
| Severe liver | 1.90 (1.61-2.22) | 1.37 (1.13-1.64) | -- | -- | 1.46 (1.23-1.71) |
| Pulmonary | 1.24 (1.14-1.34) | 1.08 (0.99-1.17) | -- | -- | 1.08 (1.00-1.18) |
| Paresis | 1.60 (1.31-1.94) | 1.23 (1.00-1.49) | -- | -- | 1.22 (1.00-1.49) |
| Metastatic | 1.47 (1.30-1.65) | 1.11 (0.97-1.28) | -- | -- | 1.12 (0.97-1.28) |
| SOT | 1.74 (1.50-2.02) | 1.12 (0.95-1.33) | -- | -- | -- |
| Charlson index | 1.08 (1.07-1.10) | **--** | 1.05 (1.04-1.06) | -- | -- |
| Admission in preceding 90 days | 1.83 (1.68-1.99) | 1.36 (1.23-1.49) | 1.41 (1.29-1.55) | 1.46 (1.33-1.60) | 1.36 (1.25-1.50) |
| Swine CAFO present | 1.15 (1.05-1.25) | 1.06 (0.97-1.16) | 1.07 (0.98-1.16) | 1.07 (0.98-1.17) | 1.07 (0.98-1.16) |
| ^a^Charlson comorbidity index.  ^b^Parsimonious model developed by stepwise selection using Akaike information criterion.[1] | | | | | |

**Table S4:** **Risk Modeling for Community-Onset *C. difficile* Infection – Sensitivity Analysis 4: Inclusion of Population Density as a Geospatial Risk Factor**

| Covariate | Univariate (Unadjusted) Odds Ratio  (95% CI) | Multivariate (Adjusted) Odds Ratio  (95% CI) |
| --- | --- | --- |
| Age, per 10 years | 1.09 (1.07-1.12) | 1.04 (1.01-1.06) |
| High-risk antibiotic receipt | 1.36 (1.23-1.50) | 1.25 (1.15-1.37) |
| Prior *C. difficile* within 90 days | 1.35 (1.00-1.82) | 1.45 (1.11-1.89) |
| Cancer | 1.36 (1.25-1.49) | 1.11 (1.01-1.21) |
| Chronic kidney disease | 1.59 (1.45-1.72) | 1.36 (1.25 -1.49) |
| Admission in preceding 90 days | 1.55 (1.42-1.69) | 1.33 (1.22-1.47) |
| Swine operation in watershed | 1.16 (1.07-1.27) | 1.07 (0.98-1.17) |
| Population density decile | 0.97 (0.95-0.98) | 0.98 (0.97-0.99) |

**References**

1.	Akaike H. Information theory as an extension of the maximum likelihood principle. Á In: Petrov, BN and Csaki, F. In: Second International Symposium on Information Theory Akademiai Kiado, Budapest, pp 276Á281, 1973.
